# Nitric Oxide in Skeletal Muscle: Role on Mitochondrial Biogenesis and Function

**DOI:** 10.3390/ijms131217160

**Published:** 2012-12-14

**Authors:** Celia Harumi Tengan, Gabriela Silva Rodrigues, Rosely Oliveira Godinho

**Affiliations:** 1 Department of Neurology and Neurosurgery, Paulista School of Medicine, Federal University of São Paulo, Sao Paulo 04039-032, Brazil; E-Mail: 1000gabi@gmail.com; 2 Department of Pharmacology, Paulista School of Medicine, Federal University of São Paulo, Sao Paulo 04044-020, Brazil; E-Mail: godinho@unifesp.br

**Keywords:** nitric oxide, nitric oxide synthase, mitochondrial biogenesis, mitochondria, mitochondrial disease

## Abstract

Nitric oxide (NO) has been implicated in several cellular processes as a signaling molecule and also as a source of reactive nitrogen species (RNS). NO is produced by three isoenzymes called nitric oxide synthases (NOS), all present in skeletal muscle. While neuronal NOS (nNOS) and endothelial NOS (eNOS) are isoforms constitutively expressed, inducible NOS (iNOS) is mainly expressed during inflammatory responses. Recent studies have demonstrated that NO is also involved in the mitochondrial biogenesis pathway, having PGC-1α as the main signaling molecule. Increased NO synthesis has been demonstrated in the sarcolemma of skeletal muscle fiber and NO can also reversibly inhibit cytochrome *c* oxidase (Complex IV of the respiratory chain). Investigation on cultured skeletal myotubes treated with NO donors, NO precursors or NOS inhibitors have also showed a bimodal effect of NO that depends on the concentration used. The present review will discuss the new insights on NO roles on mitochondrial biogenesis and function in skeletal muscle. We will also focus on potential therapeutic strategies based on NO precursors or analogs to treat patients with myopathies and mitochondrial deficiency.

## 1. Introduction

Nitric oxide (NO·) has been studied in different areas of biomedical sciences due to its multiple roles, acting as signaling molecule in several cellular processes and as a free radical, involved in oxidative damage. A simple search in PubMed (12/01/2012) using the term “nitric oxide” and “mitochondria” resulted in 2733 articles and 1940 articles were published in the last decade. These data show the great importance of this molecule in biomedical sciences with studies in different areas, aiming the elucidation of physiological pathways, pathogenesis and treatment strategies based on NO function. In fact, the use of NO in clinical practice is not new. Sodium nitroprusside, an NO donor, is a good example of clinical application that is still in use as a potent vasodilator, resulting in a rapid control of dangerous levels of arterial hypertension [1]. With the recent discoveries of other functions of NO, novel treatments may be developed in different areas of medicine, including treatment of mitochondrial abnormalities.

NO is involved in different signaling pathways in mitochondria, including control of mitochondrial respiration, apoptosis, free radical generation and more recently, mitochondrial biogenesis [2–5]. The present review will discuss the new insights of the roles of NO on mitochondrial biogenesis and function in skeletal muscle. We will also focus on potential therapeutic strategies based on NO precursors or analogs to treat patients with myopathies and other diseases associated with mitochondrial deficiency.

## 2. NO and Nitric Oxide Synthase

NO is a gaseous, inorganic, uncharged, diatomic molecule and a free radical with one unpaired electron in its external orbital [3,6]. It is highly lipophilic and diffusible, so NO can pass through multiple cell membranes to reach its final target at some distance from the sites of NO synthesis [7]. Considering its very short half-life, less than 5 s, NO cannot be stored in free form and is generally synthesized on demand with specific biological effects [7]. This molecule is produced by three isoforms of NO synthases (NOS): neuronal NOS (nNOS, EC 1.14.13.39), endothelial NOS (eNOS, EC 1.14.13.39) and inducible NOS (iNOS, EC 1.14.13.39). These isoforms were originally named because of the tissues from where they were first purified (neuronal and endothelial) and the mode of activation in the case of iNOS; but because they were also identified in several other cells, these denominations became inappropriate. Despite the proposal of other nomenclatures (numerical and descriptive) [8] the original denominations are still used. In the numerical nomenclature nNOS is type I, iNOS is type II and eNOS is type III enzyme. In the descriptive nomenclature the dependence of Ca^2+^ is indicated by a letter “c”, such as ncNOS and ecNOS, for neuronal and endothelial isoforms, respectively. These differences in nomenclature cause great confusion in the literature but in this review, we will only consider the original denominations: nNOS, eNOS and iNOS. All three types of NOS are expressed in skeletal muscle [9].

NOS uses molecular oxygen to oxidize the guanidinium nitrogen atom of l-arginine to produce NO and l-citrulline [6]. Two isoforms, nNOS and eNOS, are constitutive and regulated by the interaction of Ca^2+^ with calmodulin [6]. On the other hand, iNOS is usually only expressed during defense mechanisms against infections or inflammation [8] and it is not regulated by Ca^2+^ because it forms a complex with calmodulin at very low concentrations of Ca^2+^[2]. Four co-factors are required for NO synthesis: tetrahydrobiopterin (BH4), flavin adenine dinucleotide (FAD), flavin monucleotide (FMN) and nicotinamide adenine dinucleotide phosphate (NADPH) [3].

**Figure 1:**
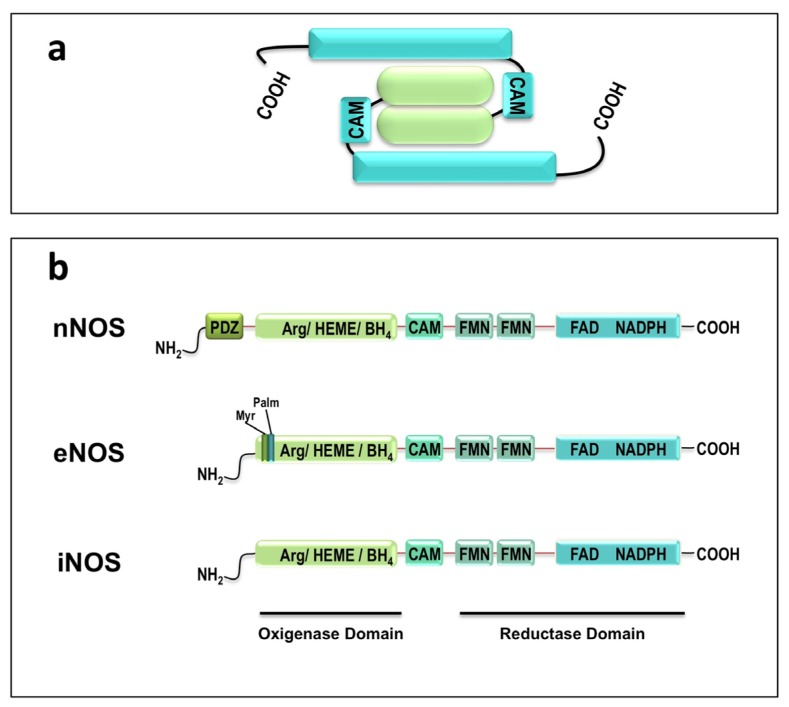
Schematic diagram illustrating the organization of human nitric oxide synthases. (**a**) Schematic figure showing the dimeric conformation of nitric oxide synthases (NOS) with both subunits attached at the oxygenase domains (green); (**b**) Main structure differences between the three types of NOS isoforms. PDZ domain is typically present in neuronal NOS (nNOS), the presence of myristoylation (Myr) and palmitoylation (Palm) sites are specific to endothelial NOS (eNOS). All isoforms the oxygenase domain contains binding sites for l-arginine (Arg), Heme and tetrahydrobiopterin (BH4) while the reductase domain binds to calmodulin (CAM), FMN, FAD and NADPH. iNOS = induced NOS.

Although all three NOS isoforms are products of different genes, they have similar structure with 50%–60% sequence identity [7] and are only active as dimer [10] (Figure 1). Each subunit of this dimeric complex is composed by two domains, oxygenase and reductase domains. The oxygenase domain contains the binding sites for l-arginine, BH4 and heme (Fe) and the reductase domain binds to FMN, FAD, NADPH and calmodulin [7,10,11]. Both subunits are attached at the oxygenase domain, forming the dimeric conformation. In skeletal muscle, the nNOS isoform has an additional 250 amino acid *N*-terminal sequence, the PDZ motif, that target the enzyme to the sarcolemma, in skeletal muscle [7]. Myristoylation and palmitoylation sites are typically present in eNOS *N*-terminal. Acylation by myristate and palmitate is required for the subcellular localization of eNOS in the caveolae of endothelial cells [11].

Since the first isolation and characterization of NOS [12], several splice variants have been discovered [13,14]. nNOS is encoded by the gene *NOS1* localized in chromosome 12q24.22, contains 29 exons and has at least ten transcript variants [15]. The classical isoform (nNOSα) is predominantly found in neuronal cells, contains both exon 2, which is responsible for the catalytic activity of the enzyme and the PDZ domain, which interacts with membrane proteins [16]. nNOSβ and nNOSδ lack the PDZ domain, maintain the catalytic domains and have different exon 1 sequence [15]. Another variant, nNOSμ, contains an additional in-frame exon between exon 16 and 17. Primarily found in mouse skeletal and cardiac muscles, it was later found in other human organs, such as aorta, bladder, colon, corpus cavernosum and placenta [17]. A human testis specific variant, TnNOS, which is analogue to the mouse nNOSδ, was also described and characterized with a shorter *N*-terminus [18].

The isoform eNOS is encoded by the gene *NOS3*, located on chromosome 7q36; and highly expressed in intestine, parathyroid, placenta, ovary, kidney, platelet and myocyte [19,20]. There are three transcript variants, with shorter distinct-*C*-terminus due to alternate 3′ exon and polyadenylation site, known as eNOS13A, eNOS13B and eNOS13C [21]. iNOS is encoded by the gene *NOS2*, located on chromosome 17q11.2 and is expressed in several cell types and tissue in response to inflammatory agents and cytokines [22]. In addition, NO can also be generated through non enzymatic sources, such as the reduction of nitrite to NO in disease states, such as ischemia, and under the acidic and highly reduced conditions [23].

### 2.1. Mitochondrial NOS

The finding of co-localization of eNOS expression with mitochondrial markers in skeletal muscle, suggested a close relationship between eNOS and mitochondria [24]. In fact, using gold labeling and electron microscopy, immunoreactivity to eNOS was found in rat liver and brain mitochondria [25]. It was, therefore, hypothesized that a mitochondrial NOS (mtNOS) could be eNOS or another NOS with significant homology to this form [25]. The co-localization of eNOS and mitochondria was demonstrated in other studies, where immunoreactivity to eNOS was found associated to skeletal muscle mitochondria in skeletal muscle [26,27]. Actually, NO production by isolated mitochondria strongly suggested the existence of an mtNOS [28,29]. Lacza *et al.* confirmed the presence of NOS activity in liver, using the assay of conversion of l-arginine to citrulline and also found immunoreactivity to eNOS by Western blot and immuno-gold labeling with electron microscopy [30]. However, the same group was not able to confirm these results in a later study, when NOS activity and NO production were not detected in liver mitochondria [31]. They also identified specific immunoreactivity to nNOS, iNOS and eNOS in liver mitochondria obtained from nNOS, iNOS and eNOS knockout mice, excluding these isoforms as the mtNOS. Studies on this subject have been controversial because they are based on the presence of NOS activity, NO production or nitrite/peroxynitrite in isolated mitochondria from rat liver [28,32,33], PC12 (rat neuronal cells) and COS-1 (monkey fibroblasts) cells [34], porcine heart [35], rat kidney [36], rat heart [37]; while others do not confirm these findings in murine liver [31,38,39]. Other studies using different approaches have suggested that mtNOS might be eNOS [25,26,40], iNOS [32,33,35,37] or even nNOS [36,41–43]. These discrepancies can be explained by differences in methods of purification of mitochondrial fraction, specificity of the method of NOS detection and different tissue/cell sources. Studies with knockout mice have also given discrepant results [31,44]. Kanai *et al.* (2001) studied heart from knockout (nNOS −/−, iNOS −/− and eNOS −/−) mice and observed absence of mitochondrial NO production only in nNOS −/− knockout mice, indicating that nNOS should be the mtNOS, specifically the nNOSα variant, which was the gene knocked out in these mice [44]. Lacza *et al*., however, have not confirmed NO production in nNOS knockout mice [45].

Only a few studies focused on the existence and identity of mtNOS in skeletal muscle [40,46]. In rat muscle, eNOS was localized in mitochondria by electron microscopy [40] which is supported by the findings of co-localization of eNOS with mitochondrial markers in rat [24] and human muscle fibers with mitochondrial proliferation [26,27]. Studying the insulin induced effects on NO synthesis in rat skeletal muscle, Finocchietto *et al.* showed an increased mitochondrial NO synthesis via Akt phosphorylation that was dimished in nNOS silenced rat muscle. Based on these results they concluded that the mtNOS would be a variant of nNOS that could be subjected to pos-translational modifications [47]. It was also suggested that nNOS could be translocated to mitochondria but with unclear mechanisms [48]. Recently, Aguirre *et al.*[46] tried to identify the nature of mtNOS performing a study on skeletal muscle mitochondria of mice injected with lipopolysaccharide (LPS), considering that mtNOS expression was reported to increase in response to endotoxin [49]. The authors observed that in the presence of a NOS substrate, mitochondria from LPS treated mice had lower respiration rate, which was prevented by a NOS inhibitor. Because they could not find the expression of the known iNOS, they suggested that the mtNOS could be an endotoxin-inducible NOS but distinct from iNOS and the other cytosolic isoforms. In skeletal muscle there are some evidences suggesting that, at least in this tissue, eNOS or a similar variant may be the mtNOS, but more clarification is still needed.

### 2.2. NOS Isoforms in Skeletal Muscle

In skeletal muscle, nNOS and eNOS isoforms are expressed constitutively while iNOS is only expressed during inflammatory responses [9,50]. Each NOS isoform has a specific localization in different compartments of muscle fibers. The nNOS isoform is considered the main source of NO in skeletal muscle, however it is not the same isoform expressed in brain. A splice variant is expressed in skeletal muscle, nNOSμ, characterized by an insertion between exon 16 and 17, encoding a 34-aminoacid segment [51]. This isoform is located on the sarcolemma (muscle membrane) [26,27], with higher activity in type II (fast twitch) fibers and linked to the dystrophin complex [26,52,53]. nNOS expression in the sarcoplasm was also reported in type I and II fibers, but more intense in type I fibers [54]. It was demonstrated that nNOSμ is also present in soluble fractions of gastrocnemius muscle homogenates in mice, showing the existence of a soluble cytoplasmic nNOSμ [55]. Studies with mice with reduced sarcolemmal nNOS but preserved soluble nNOS show that NO produced by sarcolemmal nNOS acts in the regulation of α-adrenergic vasoconstriction and blood supply in contracting skeletal muscle [55,56]. In the absence of sarcolemmal nNOS there is a considerable decrease in the blood supply during electrically evoked muscle contraction in mice [55], which demonstrates the important role of this isoform in exercise capacity. However, when sarcolemmal nNOS is absent, cytoplasmic nNOSμ is not able to compensate the NO deficiency [55]. Another nNOS splice variant, nNOSβ, was recently identified and localized in the Golgi complex [56]. The loss of nNOSμ and β in genetically modified mice showed disruption of the microtubule cytoskeleton and abnormal mitochondrial morphology, suggesting that both splice variants have an important role in maintaining normal mitochondrial health [56]. The function of the cytoplasmic nNOSμ remains unclear.

Expression of eNOS is primarily found in endothelial cells of vessels and microvessels [54] but sarcoplasmic expression has also been reported [26,27]. This pattern is confirmed by NADPH diaphorase histochemistry, which is considered an indirect method to evaluate NOS activity [26,27,53]. Abnormalities in specific isoforms such as nNOS and eNOS have been reported in muscle diseases with mitochondrial deficiencies [26,27], indicating that specific NOS activities and expression may be involved in the pathogenesis of these diseases. Increased nNOS activity and expression were observed in muscle fibers with mitochondrial proliferation, suggesting that it is related to mitochondrial biogenesis [27]. However, the exact mechanisms involved in these abnormalities are not clear.

## 3. Regulation of NOS

Due to the various physiological roles, specific tissue expression and different isoforms, it is reasonable to believe that the regulatory mechanisms controlling NOS activity, expression and localization are very complex and multifactorial. All types of NOS are transcriptionally modulated and exhibit inducible and constitutive patterns of expression in different tissue environments [7]. The mechanisms of regulation can be summarized in alternative mRNA splicing, protein-protein interactions (e.g., to Ca^2+^/calmodulin complex, PDZ domains, PIN, caveolin-1 and 3, Hsp90, ENAP-1, kalirin), covalent modifications (phosphorylation, myristoylation, palmitoylation) and redox signaling [7,11,57]. These regulatory mechanisms control the expression, localization and activity of NOS.

The expression of nNOS and eNOS in specific tissues is controlled in some cases by alternate mRNA splicing, as in the case of TnNOS, expressed in testis [18,58], while the presence of a 220 aminoacid sequence in the *N*-terminal of nNOS, containing the PDZ domain, targets nNOS to the brain and skeletal muscle [16]. In skeletal muscle, PDZ domain interactions mediate binding to syntrophin, a dystrophin associated protein, localizing the muscle nNOS to the sarcolemmal membrane. When this complex is absent, such as in Duchenne muscular dystrophy, nNOS is not attached to the membrane and localizes in the cytosol [59].

The processes of myristoylation and palmitoylation (acylation with the fatty acids myristate and palmitate) are required for the proper localization of enzyme in the caveolae of endothelial cells [60,61]. In cardiac myocytes, eNOS is localized in plasmalemmal caveolae due to the process of palmitoylation [62]. Caveolae are microdomains of the plasmalemmal membrane and it is coated by proteins called caveolin. Caveolin 1 and 3 are negative regulator of eNOS and nNOS [63].

The interaction between calmodulin and Ca^2+^ is required for nNOS and eNOS activity. However several proteins can interact and regulate the enzyme activity. The FMN-binding sub-domain can act as an enzyme inhibitor, by destabilizing calmodulin binding at low Ca^2+^ level. PIN, an 89-amino-acid protein that specifically interacts with the *N*-terminal of nNOS, has been associated with inhibition of the isoform activity [64,65], but it has also been argued that this inhibition may not be specific to nNOS [66]. The molecular chaperone Hsp90 increases eNOS activity [67] whereas kalirin, a cytosolic protein, inhibits iNOS by preventing dimer formation [68]. Structures such as BH4 and Zinc binding are not required for dimer formation but are important for stabilization of this structure, acting in the regulation of NOS activity [69]. Phosphorylation can have an inhibitory effect on nNOS activity while on eNOS the activity is increased [70–73].

The influence of redox signaling on NOS activity has been demonstrated for the endothelial isoform. ROS can affect eNOS through pos-translational modifications such as *S*-glutathionylation, *S*-nitrosylation; H_2_O_2_-dependent activation or interactions affecting signaling pathways of eNOS activation [7,57,74]. The importance of *S*-glutathionylation in the regulation of endothelial function and vascular tone has been demonstrated by the finding of increased *S*-glutathionylation in hypertensive vessels, that was restored by thiol-specific reducing agents, which reverse this *S*-glutathionylation [75].

*S*-glutathionylation induces a reversible uncoupling of eNOS, which during oxidative stress, could function to prevent irreversible oxidative damage of the thiols critical for eNOS function [76]. *S*-nitrosylation of eNOS is an important mechanism of activity control. It was demonstrated that eNOS is constitutively *S*-nitrosylated in resting endothelial cells but with the addition of an eNOS agonist, eNOS is denitrosylated and then progressively renitrosylated, resuming its original level. An inverse relationship between eNOS activity and *S*-nitrosylation was also found in endothelial cells. These results show that eNOS *S*-nitrosylation leads to a decrease in enzyme activity [77]. *S*-nitrosylation is also a dynamic mechanism of control of NOS activity. Targeting of eNOS to cellular membranes, such as caveolae, was also demonstrated as a requirement of eNOS *S*-nitrosylation while when the enzyme location in the cytosol promotes denitrosylation [77,78]. Mass spectrometry analysis showed that the location of *S*-nitrosylation is the zinc-tetrathiolate of eNOS[78]. However the roles of these mechanisms on regulation of NOS isoforms in skeletal muscle are yet to be clarified.

H_2_O_2_ is a key modulator of eNOS activation in vascular endothelial cells [79] and cardiac myocytes [80]. Using a highly sensitive fluorescent probe it was possible to demonstrate that low concentrations of H_2_O_2_ promote NO synthesis, through activation of eNOS by phosphorylation on multiple residues [80]. Comparing the effects of H_2_O_2_ treatment on cardiac myocytes from eNOS^null^ and nNOS^null^, Sartoretto *et al.* found that H_2_O_2_ treatment effects were abolished in eNOS^null^ cells while nNOS^null^ myocytes H_2_O_2_ effects were maintained, suggesting that eNOS is the main source of H_2_O_2_ induced NO synthesis [80]. Interestingly, Waypa *et al.*[81] demonstrated that H_2_O_2_ production in pulmonary smooth muscle cells under hypoxic conditions is increased in the mitochondrial intermembrane space but not in the matrix, which favours the idea of compartmentalized redox regulation.

Signaling pathways of NOS activation can also be affected by redox modifications in several intermediaries (for review see [74]).

## 4. NO and Mitochondrial Function

**Figure 2:**
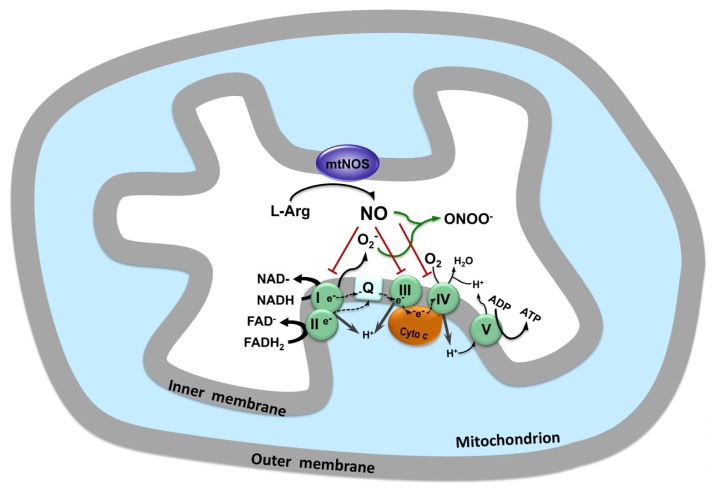
The influence of NO on mitochondrial respiratory chain. The main site of inhibition of respiratory chain by NO is at complex IV (cytochrome *c* oxidase) by competition with oxygen. NO can also inhibits the electron transport chain at complex I (NADH dehydrogenase) and III (ubiquinol cytochrome *c* oxido reductase). The disturbance in the electron transport chain favors the formation of superoxide anions (O_2_^−^). The reaction between superoxide anions and NO, results in formation of peroxynitrite (ONOO^−^) inducing macromolecular damage and cell death.

NO diffuses from mitochondria to cytosol, as well as from cytosol to mitochondria, what is called mitochondria-cytosol NO cross-talk [82]. Through the interaction with other molecules or proteins, NO exerts its function on signaling physiological events or promoting cell damage (Figure 2).

Nitrosative and oxidative modifications generally constitute redox-related signaling events in several physiological pathways or nitrosative/oxidative stress promoting mitochondrial damage [9]. NO can react to three types of targets: (a) molecular oxygen and superoxide anions; (b) transition metals such as heme iron and iron-sulfur centers; and (c) reduced thiols [83].

The reaction of NO with molecular oxygen or superoxide anions generates low molecular weight NO derivatives that participate in electron transfer reactions, as retain redox activity [84]. The formation of peroxynitrite from the combination of NO and superoxide, however, mediates oxidative damage [9].

The reaction with transition metals represents a mechanism of NO buffering or regulation. Protein activity can be modulated through reversible reaction with transition metals and thiols [84]. The interaction of NO with heme iron of soluble glanylyl cyclase induces a conformational change activating this enzyme and increasing cyclic guanosine monophosphate (cGMP) levels [84,85]. This increase activates cGMP-dependent protein kinases or cGMP-gated ion channels, characterizing the NO mediate physiological actions [82].

NO can also interact with other heme-containing proteins, such as, cytochrome *c* oxidase, or with iron-sulfur clusters present in complex I of the respiratory chain, both promoting enzyme inhibition [9].

NO reacts with protein thiol (RSH, RS-) via *S*-nitrosylation to form RS–NO groups, this reaction is reversed by NO transfer to other sulphur centers. *S*-nitrosylation of glutathione and other non-regulatory thiols can act as NO buffering and regulate protein function by altering conformation, by accelerating disulphide formation, or by influencing the reactivity of nearby metal centers [86].

Finocchietto *et al.*[47] demonstrated the role of NO on glucose metabolism. These studies demonstrated that insulin increased muscle oxidative rate via mitochondrial NO, resulting in decline of mitochondrial O_2_ uptake. In the absence of NO, mitochondrial O_2_ uptake is completely released leading to a preferential oxidation of glucose to CO_2_ and H_2_O, while in the presence of NO, glucose utilization is delivered to glycogen synthesis.

### 4.1. Control of Mitochondrial Respiration

NO affects mitochondrial function in many ways. Because of its vascular smooth muscle relaxing effect, NO regulates blood flow to skeletal muscle fiber, facilitating the supply of respiratory substrates to mitochondria [9]. NO also regulates binding and release of O_2_ from hemoglobin in red blood cells [87], thereby regulating oxygen delivery to tissues and consequently the O_2_ supply to the mitochondria [88]. The classical concept of control of mitochondrial respiration is based on the mitochondrial metabolic states, which is controlled by ADP availability [89]. In state 4, there is availability of respiratory substrate but not of ADP, while in state 3, both respiratory substrate and ADP are available. State 3 corresponds to the maximal physiological rate of ATP production and O_2_ consumption. Under non-stimulated physiological conditions only 35% of the ATP-producing capacity is used [90]. Boveris *et al.* proposed a new concept of regulation of cellular respiration that depends on energy demands, which is controlled by the availability of ADP to F1-ATPase and of O_2_ and NO to cytochrome *c* oxidase [3].

The influence of NO on mitochondrial respiratory chain can be summarized as follow: (a) NO inhibits cytochrome *c* oxidase activity by competing with oxygen; (b) NO inhibits electron transfer between cytochrome *b* and *c* and increases mitochondrial production of O_2_^−^; and (c) NO inhibits electron transfer and NADH-dehydrogenase function in Complex I [3].

### 4.2. NO Inhibits Cytochrome *c* Oxidase Activity

NO was first recognized as an inhibitor of mitochondrial electron transfer in 1994 [91,92], with the observations that low NO concentrations was able to reversibly inhibit brain and muscle cytochrome *c* oxidase through competitive binding at O_2_ site. This NO binding occurs at physiological concentrations of NO and inhibits the electron flow in the respiratory chain and therefore decreases O_2_ consumption and ATP formation [2,93]. Differently from O_2_, NO reversibly binds to both reduced and oxidized cytochrome *c* oxidase. At low O_2_ concentration, NO binds to the heme *a3* domains of reduced form of cytochrome *c* oxidase, as a competitive inhibitor of O_2_. However, at high O_2_ level, NO binds to the oxidized cytochrome *c* oxidase, via the copper moiety of the binuclear center instead of the iron moiety. This reaction results in nitrite (NO^2 −^) formation and consequently in O_2_ consumption [94]. A persistent inhibition of cytochrome *c* oxidase can promote, at an early stage, the release of small amount of hydrogen peroxide, acting as cellular signaling defense molecule. But at a later stage, high concentrations of hydrogen peroxide may lead to peroxynitrite generation and induce apoptosis or necrosis [82].

### 4.3. NO Inhibits Electron Transfer between Cytochrome *b* and *c*

Another NO sensitive site in the respiratory chain is the electron transfer at complex III, ubiquinol-cytochrome *c* reductase [3,90]. NO is able to inhibit succinate-cytochrome *c* reductase and NADH-cytochrome *c* reductase, with similar effect on both reductases but in a lower extent than cytochrome *c* oxidase [95]. Inhibition at this site increases the production of O_2_^−^ and H_2_O_2_, which are not only involved with oxidative damage, but together with NO, are considered as part of an integrated system of mitochondrial signaling for cellular regulation [82].NO is also able to reduce cytochrome *b*, probably by its interaction with an iron-sulfur center, which is reversible but is not affected by the O_2_/NO ratio [3].

### 4.4. NO Inhibits Electron Transfer and NADH-Dehydrogenase Function in Complex I

It has been shown that prolonged exposure of NO also leads to inhibition of complex I activity in murine macrophage cultured cells, with a concomitant decrease in the content of intracellular reduced glutathione [96]. At first this inhibition is reversible but it becomes persistent with time. The mechanisms of complex I inhibition are not completely clear. There are a few hypothetical mechanisms but three are the most likely: *S*-nitrosation, tyrosine nitration and damage to Fe–S centers [97]. *S*-nitrosation was suggested as an important mechanism of complex I inhibition because the inhibition was reverted by either light or reduced thiols, which are treatments that typically revert *S*-nitrosation [96,98]. Rat mitochondria from different tissues such as heart, liver and brain, exhibited inhibition of complex I after prolonged exposure to NO. The same effect was obtained after addition of peroxynitrite with tyrosine nitration, suggesting that tyrosine nitration induced by peroxynitrite would be one of the mechanisms involved in complex I inhibition [99]. This idea is supported by the finding of tyrosine nitration in Complex I subunits of bovine and human mitochondria after exposure to peroxynitrite [100].

It was hypothesized that inhibition of complex I, II and aconitase induced by NO could be caused by damage to iron-sulfur clusters and release of iron from these centers. NO would react directly with the iron, displacing elemental sulfur or cysteine residues [97]. However, Pearce *et al.* (2005) did not find any effect of NO and peroxynitrite on the cofactors of complex I and III (hemes, iron-sulfur cluster or flavin) [101]. The same result was found with Complex II, with the exception of the cofactor in aconitase, thus it was suggested that mammalian mitochondrial iron-sulfur cluster are resistant to degradation from oxidative/nitrosative stress [97,101,102].

## 5. NO and Mitochondrial Biogenesis

NO plays an important role in mitochondrial biogenesis of skeletal muscle [88]. Several studies have shown that treatment of cells with NO donor increase mitochondrial markers, demonstrating induction of biosynthesis of functional mitochondria able to generate ATP via oxidative phosphorylation [5,103,104]. Treatment of rat primary skeletal muscle cultures with the NO donor *S*-nitroso-*N*-acetylpenicillamine (SNAP) led to a significant increase in mitochondrial content [27], a phenomenon also observed in other tissues. For example, studies performed in the primary cultures of brown adipocytes, which express a larger number of mitochondria than other cell types, have shown that incubation with SNAP increases the mitochondrial size and mitochondrial DNA (mtDNA) content in a concentration dependent manner [4]. The inductive effect of SNAP was completely abolished by supplementation of the medium with the NO scavenger oxyhemoglobin, indicating that the stimulatory effect depends on generation of NO.

**Figure 3:**
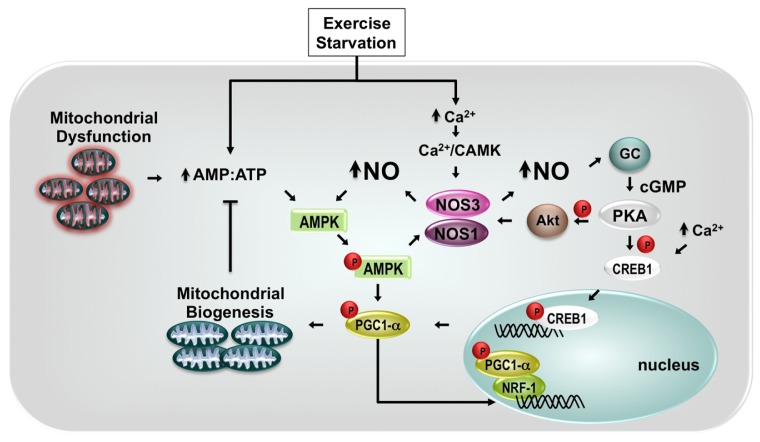
Schematic diagram illustrating the major NO pathways in the activation of mitochondrial biogenesis. Intracellular calcium release activates calcium/calmodulin kinase II (CaMK) triggering sequential activation of NOS and guanylate cyclase (GC) to generate cyclic GMP, which in turn activates protein kinase A (PKA). PKA phosphorylates CREB1 allowing its nuclear translocation and activation of the *PGC-1* gene (peroxisome proliferator-activated receptor gamma co-activator 1), a co-activator for NRF-1 (nuclear respiratory factor-1), a transcription factors for mitochondrial biogenesis. The NOS-dependent induction of mitochondrial biogenesis also involves activation of AMP-activated kinase (AMPK), allowing phosphorylation of PGC-1.

The exact molecular pathways involved in mitochondrial proliferation in response to NO signaling are not entirely understood, but in many tissues and cells, they have been associated to peroxisome proliferator-activated receptor gamma coactivator-1 (PGC-1α) [5,103,104] (Figure 3). In brown adipocyte tissue, NO-induced mitochondrial biogenesis is mimicked by incubation of cells with 8-Br-cGMP, indicating that it depends on activation of guanylil cyclase and generation of guanosine 3′,5′-monophosphate (cGMP). The downstream mechanism requires induction of PGC-1α expression since PGC-1α antisense oligomers reduced the SNAP-dependent increase in mtDNA content [4]. In addition, ectopic expression of PGC-1α into skeletal muscle cell lineage C_2_C_12_ induces a 2-fold increase in mtDNA content which was associated to a parallel increase (~60%) in mitochondrial density (mitochondrial number/cytoplasmic area) [105]. The PGC-1α-dependent increment in mitochondrial biogenesis occurs in parallel with increase in the basal oxygen consumption and in the expression (transcripts/proteins) of respiratory chain components, including the nuclear-encoded cytochrome *c* oxidase subunit IV and the mitochondrial-encoded cytochrome *c* oxidase subunit II and cytochrome *c*[105].

Stimulation of mitochondrial biogenesis by NO also requires the expression of transcriptional factors, that include CREB1 (cAMP response element-binding protein 1) and the nuclear respiratory factors 1 and 2 proteins (NRF-1 and NRF-2), which mediate expression of multiple nuclear genes encoding for mitochondrial proteins, such as the mitochondrial rate-limiting enzyme for heme biosynthesis, 5-aminolevulinate synthase [106] and, the subunits of the respiratory chain complexes [107].

This process involves an orchestrated cascade of phosphorylation events performed by distinct serine/threonine kinases. For example, while both serine/threonone kinases Akt [108] and PKA (cyclic AMP-dependent protein kinase) are able to activate eNOS via phosphorylation of Ser1177, leading to increased production of NO, phosphorylation of CREB transcription factor by PKA results in increased expression of PGC-1α, which in turn increases mitochondrial biogenesis. Actually, the most potent activator of PGC-1α transcription is the coactivator of CREB, named TORC1 (transducer of regulated CREB binding protein 1). According to Wu *el al.* (2006) [109], TORC 1, 2 and 3 markedly increases PGC-1α promoter activity, in a CREB-dependent fashion in primary mouse skeletal muscle cultures. The authors have shown that transduction of mouse primary myotubes with an adenovirus expressing hTORC1, hTORC2, hTORC3 resulted in increased mRNA levels of PGC-1α and mitochondrial markers *Cyt c*, *Cox II*, and *IDH3*α (isocitrate dehydrogenase 3α). In addition, Akt may form a physical complex with PKA serving as a substrate of PKA [110]. Once phosphorylated, Akt activates eNOS, leading to increased NO production. Conversely, Akt may induce mitochondrial biogenesis through the phosphorylation of NRF-1 and CREB1, enabling their nuclear translocation and activation of target genes, such as mitochondrial transcription factor A (Tfam), which is required for mtDNA transcription and replication [111] (for review, see [112]).

The molecular mechanisms of NO-dependent regulation of PGC-1α/ mitochondrial biogenesis also involve 5′-AMP-activated protein kinase (AMPK), a heterotrimeric kinase allosterically activated by AMP and inhibited by ATP, which serves as an energy sensor, under conditions of low energy charge (decrease in ATP and increase in AMP). Thus, conditions that cause significant cellular energy stress, such as exercise, starvation or mitochondrial dysfunction are able to increase AMPK activity (for review, see [113]) and promote mitochondrial biogenesis [114–116]. For example, exercise training performed in rat or humans increases mitochondrial content and capacity [117–119]. In fact contraction induced by electrical stimulation of isolated rat skeletal muscle also increases NO release from muscle [120] with a significant contribution of both calcium-calmodulin-dependent isoforms of NOS, nNOS and eNOS [121]. In working tissues, such as skeletal and cardiac muscle, increased levels of intracellular calcium during contraction have been associated to physiological induction of mitochondrial biogenesis. Intermittent exposure of L6 myotubes to the Ca^2+^ ionophore ionomycin (5 h/day) or to caffeine or W7, which release Ca^2+^ from the sarcoplasmic reticulum induced an increase in mitochondrial enzymes [122]. Interestingly, skeletal muscles from transgenic mice that selectively express a constitutively active form of calcium/calmodulin-dependent protein kinase IV showed augmented mtDNA replication and mitochondrial biogenesis. These effects are associated to up-regulation of mitochondrial enzymes involved in fatty acid metabolism and electron transport, and reduced susceptibility to fatigue during repetitive contractions. In mice C2C12 myotubes, the effect of CaMK was associated to activated the PGC-1α gene promoter and increased expression of PGC-1α [123]. Thus, calcium-regulated signaling pathways are able to induce mitochondrial biogenesis via activation of NOS or stimulation of Ca^2+^-dependent transcription factors. Interestingly, during exercise in humans, AMPK phosphorylates skeletal muscle nNOSμ at Ser1451 [124], which may account for the increase in glucose uptake during exercise. Indeed, taking into account that AMPK is able to phosphorylate and activate eNOS/nNOS during contracting human skeletal muscle [125], these data suggest that phosphorylation of NOS by AMPK may be, at least in part, involved in mitochondrial biogenesis in muscle cells.

This correlation of AMPK, PGC-1α, NO and mitochondrial biogenesis in L6 myogenic cell lineage was recently confirmed by McConell *et al.*[103]. Incubation of L6 myotubes with AICAR, which activates AMP-activated protein kinase (AMPK), increased myotube mitochondrial biogenesis. The AMPK activator also increased the expression of PGC-1α, subunits I and IV of cytochrome *c* oxidase. Actually, while SNAP increased phospho-AMPK and markers of mitochondrial biogenesis (PGC-1α, NRF-1, COX-1 and COX-4), inhibition of NOS with l-NAME attenuated the AICAR-induced increases in COX-1 and COX-4 protein, indicating that activation of AMPK in L6 myotubes increases mitochondrial biogenesis, at least in part, via interactions with NOS.

## 6. NO and Myogenic Differentiation

Differentiation of embryonic muscle cells involves fusion of mononucleated myoblasts derived from pluripotent mesodermic cells to form multinucleated myotubes. Myotubes differentiate into mature skeletal muscle fibers with functional contractile apparatus and compartmentalized expression of synaptic proteins [126]. Beyond this stage, mitosis and DNA synthesis arrest almost completely and generation of new fibers depends on quiescent precursor cells named “satellite cells”, located under the basal lamina of individual postnatal and adult myofibers.

Both embryonic and adult myogenic cells are influenced by changes in NO levels. While embryonic myoblasts have NOS activity, as measured by their ability to convert arginine to citrulline [127], adult satellite cells are activated by nitric oxide (NO) produced in muscle fibers, which may contribute to post-injury regeneration of skeletal muscle. According to Lee *et al.*[127] NOS activity dramatically increases in chick embryonic myoblasts that are competent for fusion, and coincides with increment in intracellular cGMP levels. The authors also showed that while NO donor SNP (sodium nitroprusside) accelerated myoblast fusion, the NOS inhibitor l-NMMA (NG-methyl-l-arginine) delayed the fusion that is reversed by treatment with 8-Br-cGMP, an analog of cGMP.

Differentiation of myogenic cell also requires mitochondrial elongation that depends on the cellular generation of NO. Using myogenic precursor cells isolated from the muscles of newborn mice, De Palma *et al*. [128] showed that inhibition of NO synthesis lead to inhibition of both mitochondrial elongation and myogenic differentiation. This phenomenon was associated with NO-dependent inhibition of dynamin-related protein-1 (Drp1) [128], a GTPase protein that induces mitochondrial fission and, as consequence, interferes with maintenance of mitochondrial integrity (for review see [129–131]. By inhibiting Drp1, NO increases the formation of mitochondrial network, due to the ongoing mitochondrial fusion, and stimulates myogenic differentiation.

A better understanding of NO interference on myogenic differentiation may be of crucial importance to development of new therapeutic strategies for enhancing skeletal muscle mass and performance, opening promising perspectives for treatment of conditions associated with reduced proliferation of myogenic cells, such as aging or degenerative diseases.

## 7. Potential Therapeutic Strategies

Several drugs with NO-donating properties are currently in use in clinical practice, mostly for cardiovascular diseases, such as nitrovasodilator, drugs affecting NOS mRNA and/or protein levels with special modulation of eNOS [7]. A specific treatment of muscle diseases based on NO physiology is still not available, however several strategies have been studied in recent years. Focusing on skeletal muscle and mitochondrial dysfunction, NO-directed approaches can be summarized in: diet, exercise and pharmacological approaches to increase NO in subcellular compartments [132]. The main objective when focusing on mitochondrial deficiencies is to increase the energy supply by improving the oxidative phosphorylation efficiency or increasing mitochondrial content.

It has been shown that calorie restriction lowers whole-body energy expenditure and induces mitochondrial biogenesis in overweight non-obese humans [133]. This study also demonstrated a decrease in DNA damage and proposed that caloric restriction would induce biogenesis of “efficient” mitochondria in human skeletal muscle as an adaptive mechanism, reducing oxidative stress. Calorie restriction was also able to increase mitochondrial biogenesis, eNOS expression and Akt phosphorylation in mice skeletal muscle, indicating that the NO pathway is activated [134].

Several studies using rodent models have shown that exercise training induces an increase in skeletal muscle mitochondrial content [135], which was demonstrated by increased expression of mitochondrial markers. As discussed in section 5, the mechanism of exercise induction of mitochondrial biogenesis involves the PGC-1α pathway [136]. It is suggested that the source of NO in exercise induced mitochondrial biogenesis is eNOS, as Nisoli *et al*. (2004) [104] reported that eNOS −/− mice had reduced mitochondrial content in skeletal muscle. However, Wadley *et al.* (2007) could not confirm the involvement of either eNOS or nNOS in the exercise induced mitochondrial biogenesis, studying eNOS −/− and nNOS −/− mice [135]. Later, Lee-Young *et al.* (2010) showed that eNOS is critical to muscle cell signaling during exercise *in vivo*, by showing that ablation of eNOS in eNOS −/− mice resulted in impaired exercise capacity [137]. Studies evaluating the exercise training in patients with mitochondrial myopathy, a genetic disorder with oxidative phosphorylation deficiency, aim the stimulation of mitochondrial biogenesis and a gene shifting, with increase of mitochondrial content with normal mtDNA [138–140]. In fact, improvement of muscle strength and oxidative capacity have been demonstrated after exercise training [140] but further investigations are still needed to determine the safety, effective benefits and the most appropriate exercise training protocol.

Supplementation with arginine or citrulline has also been proposed for treatment of mitochondrial disorders with NO deficiency [141]. l-arginine was first used in the treatment of a mitochondrial encephalopathy with stroke like syndrome (MELAS) as an NO precursor with the objective to promote vasodilation in cerebral vessels [142,143]. The rationale of this approach is that patients with MELAS probably have a deficiency in smooth muscle vascular relaxation, which could be responsible for the symptoms of cerebral ischemia. This therapeutic approach is supported by the finding of low levels of serum arginine [142], low levels of citrulline [144] and endothelium dysfunction [145] in patients with MELAS. This treatment showed promising results with significant decrease in stroke frequency, improvement of neurological deficits [142,143,145,146] and increased NO production [147]. Based on these findings and the observation of NO deficiency in muscle fibers with cytochrome *c* oxidase deficiency of patients with mitochondrial myopathy [27], it was proposed that arginine and citrulline supplementation could have a broader application in mitochondrial diseases, especially in those with muscular symptoms [141].

Mitochondrial biogenesis can also be activated with the use of drugs, such as resveratrol (3,5,4′-trihydroxystilbene), a diet-derived polyphenol. Treatment of rodents with resveratrol induced increased mitochondrial content and improved function in skeletal muscle [148,149]. The mechanism of action involves the decrease in PGC-1α acetylation with resulting increase in PGC-1α activity [148] and AMPK activation [150]. Studies using endothelial cells demonstrated that resveratrol induces an up-regulation of eNOS [151]. Other drugs acting specifically on eNOS have been largely studied in cardiovascular diseases [132]. One example is statin that is classically known by the effects on dyslipidemia, however other beneficial pleiotropic effects have been reported [132]. Statin pleiotropic effects are mainly reported in studies with endothelial cells showing that eNOS activity may be increased [152]. However, it is well known that statins, such as sinvastatin and atorvastatin, can induce myopathy with rhabdomyolysis. A recent study in mice has demonstrated that the treatment with a novel class of statin, an NO-donating atorvastatin was able to prevent the atorvastatin induced skeletal muscle dysfunction [153]. The authors showed that the NO-donating atorvastatin maintained the lipid lowering effect with absence of the other side effects observed with atorvastatin, such as sarcolemmal damage and mitochondrial dysfunction.

NO-donating drugs have also been tested in Duchenne muscular dystrophy in order to improve muscle regeneration and restore NO intracellular level, which is diminished due to the loss of nNOS. Brunelli *et al.*[154] studied the treatment with HCT 1026, a derivative of flurbiprofen, a nonsteroidal anti-inflammatory drug, that releases NO and with no severe side effects. They showed that HCT 1026 slowed down disease progression and ameliorated the morphological, biochemical and functional phenotype. In the same study arterially delivered donor stem cells in association with HCT 1026 presented an enhanced therapeutic efficacy. This treatment demonstrated better results than the combination of l-arginine and deflazacort [155]. Recently, Buono *et al.*[156] demonstrated a therapeutic effect of molsidomine, another NO releasing drug, in dystrophic mice. This treatment increased regenerating muscle fibers, as well as led to functional amelioration of the muscle, due to increased proliferation of satellite cells. These studies open up a new possibilities for treatment of muscle diseases including those with mitochondrial dysfunction, especially because they are drugs already tested in humans and could be used in long term treatments.

## 8. Conclusions

NO has important roles on the regulation of mitochondrial function and induction of mitochondrial biogenesis. The pathways involved in NO signaling are complex but an increasing number of articles have addressed this subject in recent years. Several therapeutic approaches have been proposed with the aim of increasing mitochondrial content to compensate mitochondrial deficiency and to increase energy supply. However a lot of work has to be done to elucidate the exact mechanisms involved to make this approaches viable for clinical practice.
